# Evaluation of hTERT, KRT7, and survivin in urine for noninvasive detection of bladder cancer using real‐time PCR

**DOI:** 10.1186/s12894-021-00838-z

**Published:** 2021-04-19

**Authors:** Reza Yahyazadeh, Davood Bashash, Parisa Ghaffari, Saeid Kord, Ava Safaroghli-Azar, Seyed H. Ghaffari

**Affiliations:** 1 Department of Urology, Shariati Hospital, Tehran University of Medical Sciences, Tehran, Iran; 2 Department of Hematology and Blood Banking, School of Allied Medical Sciences, Shahid Beheshti University of Medical Sciences, Tehran, Iran; 3 Hematology, Oncology and Stem Cell Transplantation Research Center, School of Medicine, Shariati Hospital, Tehran University of Medical Sciences, Tehran, Iran

**Keywords:** Bladder cancer, Transitional cell carcinoma, hTERT, KRT7, Survivin, Urine cytology, Real‐time PCR

## Abstract

**Background:**

Transitional cell carcinoma (TCC) of the bladder is the second most common genitourinary malignancy. Because of the low sensitivity of urinary cytology and the invasiveness and expense of frequent cystoscopies for the detection of low-grade superficial lesions, we aim to establish a sensitive molecular approach to detect bladder cancer noninvasively.

**Methods:**

Voided urine samples were collected from 80 patients with bladder cancer at the time of diagnosis, in addition to 30 patients with non-bladder cancer urological diseases and 20 healthy volunteers. The level of hTERT, KRT7, and survivin (SVV) mRNAs were analyzed using a qRT-PCR assay.

**Results:**

The optimal threshold values for hTERT, KRT7, and SVV in urine were calculated by ROC curves analysis. The overall sensitivity was 81.3%, 91.3%, and 68.8% for hTERT, KRT7, and SVV, respectively, which were significantly higher than urine cytology (22.2%, p < 0.001). A higher positive ratio was obtained using multi-marker detection in comparison to single marker detection. The combined use of markers increased the sensitivity of cytology from 22.2 to 100%. In contrast with the urine cytology method, the sensitivity of these biomarkers was not correlated with the grades and stages of the bladder tumors.

**Conclusions:**

Our data indicate that urinary hTERT, KRT7, and SVV have superior sensitivities over cytology. The combined use of these markers offers a powerful potential assay and promising tool for a sensitive, noninvasive, and highly specific diagnostic method and follow-up of low-grade TCC of the bladder.

## Background

Transitional cell carcinoma (TCC) of the bladder is the second most common malignancy of the genitourinary tract and the third most common cause of death among people with genitourinary tumors [1]. TCC responds well to local resection and subsequent adjuvant intravesical treatment [2]. Nevertheless, the recurrence rate of TCC is 50–70%. While 10% of pTa and 30% of pT1 tumors progress to muscle-invasive disease, a majority (80%) of the cases present with non-muscle-invasive papillary tumors (stages pTa or pT1), which have a much more satisfactory prognosis [3].

The heterogeneous characteristics, diverse genetic architecture coupled with different clinical manifestations put unresolvable obstacles in the way of successful diagnosis of bladder cancer [4]. From the first description of bladder cancer, the silent clinical manifestation of the disease, especially in low-grade stages, was one of the main challenges that physicians confronted [5]. Of note, the longer the diagnosis is delayed, the greater the risk of metastasis, which in turn would alter non-lethal cancer to a life-threatening malignancy [6, 7]. This unique feature highlights the importance of applying accurate as well as effective strategies to diagnose bladder cancer, not only rapidly but also with acceptable sensitivity. As the list of the proposed techniques for the detection of this malignancy is continually growing, interest in applying more accurate and affordable methods has increased overwhelmingly. Apart from cystoscopy and urine cytology, which are the gold standard techniques for the diagnosis of this cancer [8], recent molecular investigations have declared the rewarding impact of mRNA expression analysis not only in the detection but also in the follow-up of patients with bladder cancer [9–11]. Attention in recruiting this method has emerged from recent disclosures indicating the remarkable results in both its sensitivity and specificity, which matters especially in low-grade patients [12]. Besides its accuracy, an appealing advantage to this technique is the possibility of examination of the urine specimen, which categorizes it as a non-invasive approach [12]. By opening a valuable avenue for bladder cancer detection, identifying a group of genes that can be exploited for better diagnosis is still a debatable issue. Wide varieties of genes with different functions have been suggested to evaluate urine samples of bladder cancer patients [13–15]. However, some of these genes lost their importance in clinical investigations due to their lack of sensitivity or correlation with the stage of the disease.

There is a pressing need for a non-invasive method to diagnose carcinoma of the urinary bladder. Invasive cystoscopy examination remains the gold standard; nonetheless, it is required not only for the diagnosis but also for the repeated 3-month follow-up intervals. This is due to the fact that no currently available method is adequately sensitive and specific [16]. A method that could replace cystoscopies or at least reduce their number in given situations as well as adhering to greater accuracy than cytology would be highly commended by both patients and clinicians. Therefore, identification of urinary biomarkers for the detection of bladder cancer recurrence would be beneficial to minimize the frequency of cystoscopy.

In the present study, we aim to establish a noninvasive sensitive molecular approach for the detection of bladder cancer. We sought to evaluate the diagnostic potential of measuring three molecular markers (hTERT, SVV, and Keratin7 mRNAs) in the voided urine samples from patients with primary bladder carcinomas.

## Methods

### Patients and samples collection

Eighty patients diagnosed with bladder cancer, who were admitted to the Urology Department, of Shariati Hospital, were included in this study after giving informed consent. The diagnoses were made via cystoscopy and histopathology. The standard evaluations included urine analyses, blood chemistries, and radiological assessments. Any patients who had undergone any previous treatments were excluded from this study. A group of 30 patients suffering from hematuria due to non-neoplastic causes was used as a control (urinary tract infections, stones, benign prostate hyperplasia, and combined disorders). A group of 10 healthy volunteers was also included in this study. All subjects except 10 healthy volunteers, underwent cystoscopy as a reference standard for the detection of bladder cancer, and all tumors or suspicious lesions were resected for histopathological examination. The final diagnosis of bladder cancer was based on a histological examination. Tumor staging and grading were determined according to the TNM and World Health Organization classification [17]. Voided urine was obtained from the patients before they received any treatment and before they underwent surgery. Approximately 40 ml of morning voided urine samples were collected from the patients and the samples were tested for urine cytology in addition to the detection of mRNA for the biomarker genes via quantitative Real-time RT-PCR (qRT-PCR).

### RNA isolation and cDNA synthesis

The urine samples were centrifuged and the pellet cells were washed twice with PBS. Next, RNA extraction was carried out using the FastPure RNA kit (Takara Bio, Inc., Otsu, Japan). About 1 µg of total RNA was subjected to reverse transcription using the PrimeScript RT reagent kit (Takara Bio) according to the manufacturer’s instructions.

### Quantitative real‐time PCR

Quantitative real-time RT-PCR was performed on a light cycler instrument (Roche, Germany) using SYBR Premix Ex Taq technology (Takara Bio). PCR was conducted in a 20 µl reaction mixture including; 10 µl of SYBR Green master mix, 2 µl of cDNA samples, 0.5 µl of forward and reverse primers (10 pmol) in water plus 7 µl of nuclease-free water (Qiagen, Hilden, Germany). Thermal cycling conditions involved an initial activation step for the 30 s at 95 °C, followed by 45 cycles including a denaturation step for 5 s at 95 °C and a combined annealing/extension step for 20 s at 60 ºC. Melting curve analysis was applied to validate whether all the primers yielded a single PCR product.

The target gene expression levels were normalized to the hypoxanthine phosphoribosyl transferase1 (HPRT1) levels in the same reaction. Relative expression levels of the target genes within a sample was calculated using the 2^−ΔΔCq^ formula, ΔΔC_q_ = (C_q Target _− C_q HPRT1_) experimental sample − (C_q Target_– C_q HPRT1_) control samples, where Cq is the quantification cycle. The fold expression relative to the average calibrator ΔC_q_ value (20 samples from healthy individuals and 20 samples from patients with urological disorders other than bladder cancer) was normalized to a reference gene (*HPRT1*). Samples were classified as positive for a particular gene if the 2^− ΔΔCq^ was above the cut-off point. The validity of qPCR products was confirmed by gel electrophoresis and sequencing of the representative qPCR reactions (data not shown). The sequences of the gene-specific primers are summarized in Table 1.

**Table 1:** Nucleotide sequences of primers used for real-time RT-PCR

Gene	Forward primer (5′–3′)	Reverse primer (5′–3′)
hTERT	TGACACCTCACCTCACCCAC	CACTGTCTTCCGCAAGTTCAC
SVV	CCAGATGACGACCCCATAGAG	TTGTTGGTTTCCTTTGCAATTTT
KRT7	TGTGGATGCTGCCTACATGAGC	CAATCTCCTGCTTGGTGTTGCG
HPRT1	TGGACAGGACTGAACGTCTTG	CCAGCAGGTCAGCAAAGAATTTA

### Statistical analysis

Nonparametric receiver operating characteristic analysis (ROC), an area under the curve (AUC), sensitivity, specificity, as well as likelihood ratios were calculated to determine the levels of hTERT, SVV, and KRT7 biomarkers that best differentiate the bladder cancer cases from the control subjects. The optimal cut-off values were calculated as the marker level that maximized the sensitivity and specificity. The likelihood ratio was calculated based on the following formula: LR+ = sensitivity/1 − specificity. To evaluate the performance of the biomarkers in the voided urine samples from bladder cancer patients, we computed the sensitivity, specificity, positive predictive value (PPV), negative predictive value (NPV), and the accuracy for cytology, hTERT, KRT7, and SVV when tested independently or in combinations in the urine samples. The comparison of the clinicopathological factors was analyzed using the Student’s t test, chi-square tests, and ANOVA. All the performed statistical tests were two-sided and the *P*-values of < 0.05 were considered to be of statistical significance. The statistical analyses were performed using the Statistical Package for the Social Sciences (SPSS 21, Chicago, IL).

## Results

### Patients

Of the 80 patients with bladder cancer enrolled in this study, there were 20 females and 60 males, with a median age of 63 years (age range 31–81 years). From 20 patients with urological disorders other than bladder cancer and 10 healthy volunteers, there were 10 females and 30 males with a median age of 58 years (range 28–70 years). According to the pathological report of surgical specimens, the tumor was low-grade in 60 (75%) and high-grade in 20 (25%) patients. The stage of cancer was Ta in 50 (62.5%), T1 in 17 (21.25%), T2 in 7 (8.75%), and T3 in 6 (7.5%). Voided urine cytology was positive in 25 (31.3%) and negative in 56 (70.0%) (Table 2).

**Table 2:** Clinicopathologic characteristics of bladder cancer (80 patients)

Characteristics	No. pts
*Sex*	
Male	60 (75%)
Female	20 (25%)
Age; median (range), years	63 (31–81)
*Grade*	
Low	60 (75%)
High	20 (25%)
*Stage*	
Ta	50 (62.5%)
T1	17 (21.25%)
T2	7 (8.75%)
T3	6 (7.5%)
*Cytology*	
Pos	25 (31.3%)
Neg	56 (70.0%)
*hTERT*	
Pos	65 (81.2%)
Neg	15 (18.8%)
*KRT7*	
Pos	73 (91.3%)
Neg	7 (8.8%)
*SVV*	
Pos	58 (72.5%)
Neg	22 (27.5%)

### Overall sensitivity, specificity, PPV, and NPV

**Fig. 1:**
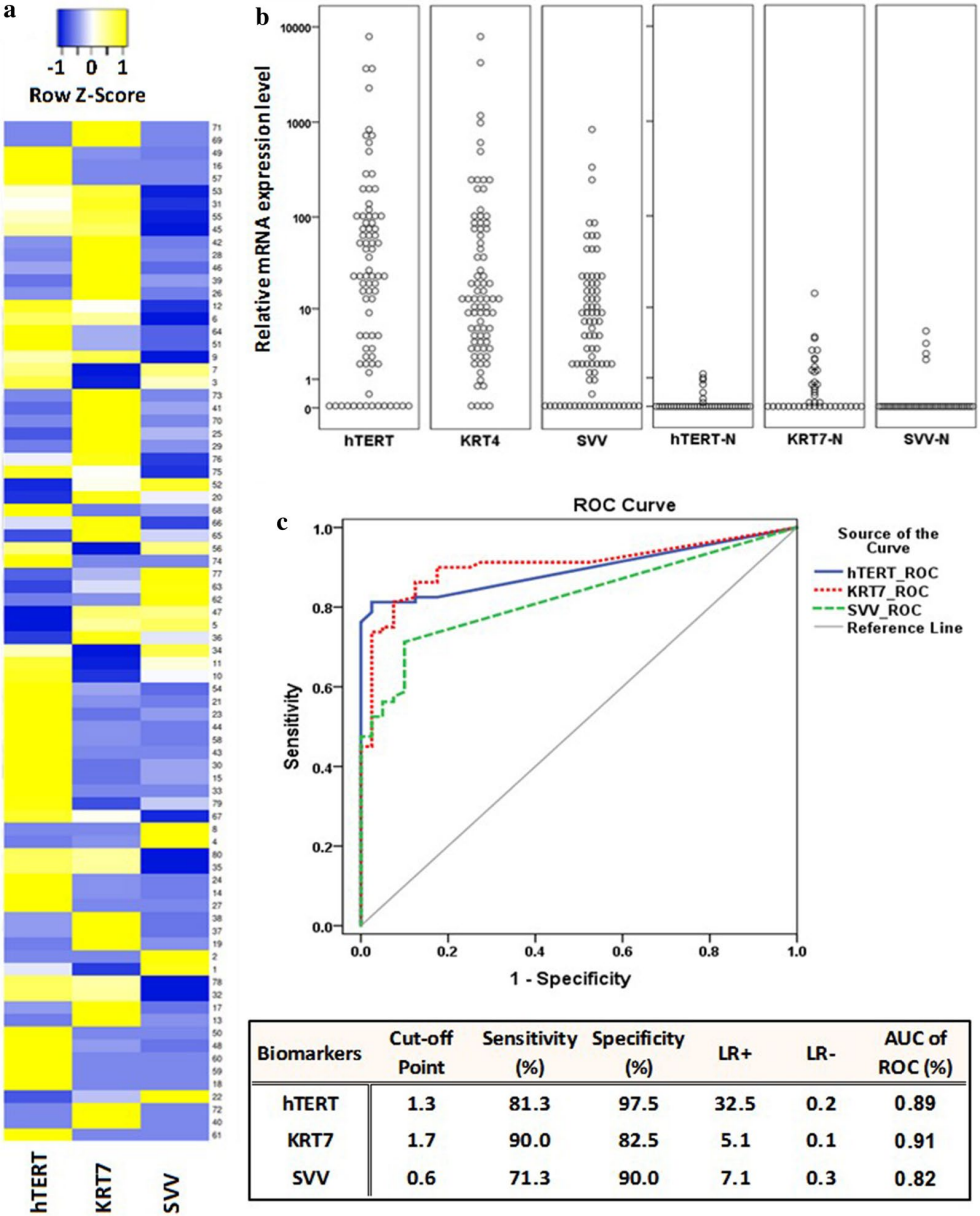
Expression levels of hTERT, KRT7, and SVV mRNA in 80 bladder cancer patients.** a** Heat map representation of the three mRNA expression levels in the 80 bladder cancer urine samples. **b** Comparison of hTERT, KRT7, and SVV mRNA levels in the urine samples of bladder cancer and non-bladder cancer patients. **c** Receiver operating characteristic (ROC) curves were performed to determine the area under the ROC curve (AUC), sensitivity, specificity, as well as the likelihood ratios (LR^+^ and LR^−^) to determine the levels of the biomarkers that best differentiate the bladder cancer cases versus the control subjects. The optimal cut-off values were calculated as the marker level that maximizes the sensitivity and specificity

The biomarkers gene expression levels in the urine samples were determined using a relative quantification method. To quantify the relative level of mRNA in urine samples, the C_q_ values of all samples were normalized to the Ct value of HPRT1 transcripts. Then, fold changes were calculated relative to the average Cq value from the control groups, including 30 urine samples from patients suffering from hematuria due to non-neoplastic causes and 10 urine samples from healthy individuals. As shown in Fig. 1, the median mRNA levels of hTERT, KRT7, SVV were significantly higher in the malignant group when compared with the normal control groups (p < 0.001). Hematuria did not affect the level of markers in patients with no bladder cancer.

In an attempt to evaluate the ability of molecular discrimination between positive and negative results, we performed the Receiver Operating Characteristic (ROC) curves for each study variable. The area under curve (AUC) for hTERT (0.89), KRT7 (0.91) and SVV (0.82) was significantly larger than the reference line (*P* < 0.01) (Fig. 1). The optimal cut-off values for the investigated biomarkers were determined based on the best balance of sensitivity and specificity along with larger increases in the likelihood ratios (LR+). The sensitivity, specificity, likelihood ratio, and AUC of ROC of all the three markers in the voided urine samples are shown in Fig. 1. The optimal cutoff values were 1.3, 1.7, and 0.6 for hTERT, KRT7, and SVV, respectively. Samples were classified as positive for a particular gene if the 2^−ΔΔCq^ was above the cut-off point.

We calculated overall sensitivity, specificity, PPV, NPV, and accuracy for cytology, hTERT, KRT7, and SVV when tested independently or in combinations in voided urine samples of bladder cancer patients. As shown in Table 3, the overall sensitivity and specificity were 81.3 and 97.5% for hTERT, 91.3 and 85% for KRT7, and 72.5 and 90% for SSV. The overall sensitivity for voided urine cytology was 31.3%; however, the voided urine cytology has a higher specificity than the other markers (100%). The PPV was 98.5, 92.4, 93.5, and 100% for hTERT, KRT7, SSV, and voided urine cytology, respectively. The NPV was 72.7, 82.9, 62.1, and 42.1% for hTERT, KRT7, SSV, and voided urine cytology, respectively. The overall accuracy was higher for KRT7 than hTERT, SSV, and voided urine cytology (89.2, 87.5, 78.3, and 54.2%).

**Table 3:** Overall sensitivity, specificity, positive predictive value (PPV), negative predictive value (NPV), and accuracy for cytology, hTERT, KRT7, and SVV when tested independently or in combinations in the voided urine samples of bladder cancer patients

	Sensitivity	Specificity	PPV	NPV	Accuracy
Cytology	31.3	100.0	100.0	42.1	54.2
hTERT	81.3	97.5	98.5	72.7	87.5
KRT7	91.3	85.0	92.4	82.9	89.2
SVV	72.5	90.0	93.5	62.1	78.3
Cyt + hTERT	85.0	100.0	100.0	74.1	88.3
Cyt + KRT7	95.0	85.0	92.7	89.5	91.7
Cyt + SVV	77.5	90.0	93.7	63.2	79.2
hTERT + KRT7	100.0	85.0	93.0	100.0	95.0
hTERT + SSV	95.0	90.0	94.9	87.8	92.5
KRT7 + SVV	93.8	75.0	88.2	85.7	87.5
Cyt + hTERT + KRT7	100.0	85.0	93.0	100.0	95.0
Cyt + hTERT + SSV	96.3	90.0	95.0	90.0	93.3
Cyt + KRT7 + SSV	96.3	75.0	88.5	90.9	89.2
hTERT + KRT7 + SVV	100.0	75.0	88.9	100.0	91.7
Cyt + hTERT + KRT7 + SVV	100.0	75.0	88.9	100.0	91.7

The combination use of the markers was calculated (Table 3). The sensitivity of cytology was increased when it was combined with any other markers. The combination use of the two markers (hTERT and KRT7) gave the highest sensitivity and overall accuracy (100 and 95%), with a specificity of 85%. There was no significant difference in sensitivity of hTERT, KRT7, and SVV concerning sex, history of bladder cancer, tumor burden, and tumor stage.

### Comparison of hTERT, KRT7, and SVV detection in different grades of the tumor

**Fig. 2:**
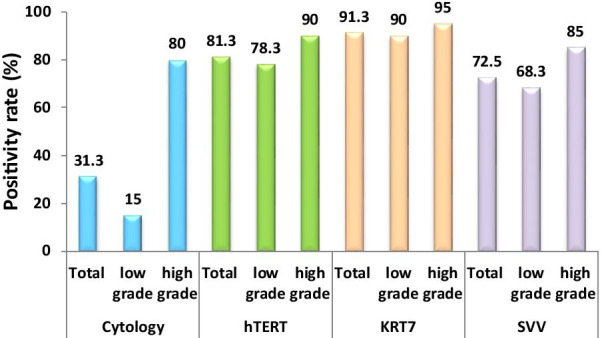
The positivity rate of urine cytology, hTERT, KRT7, and SVV in the different grades of bladder cancer

The sensitivity of these tests was studied separately in high and low-grade bladder carcinomas (Table 4; Fig. 2). According to the results, in low-grade tumors, the cytology was positive in 9 out of 60 patients (15%) and negative in the rest of the 51 patients (85%). In high-grade tumors, cytology was positive in 16 out of 20 patients (80%) and negative in the rest of the 4 patients (20%). The sensitivity of urine cytology for the diagnosis of low-grade tumors was significantly lower than that of high-grade tumors (p < 0.001). As shown in Table 4, the result of the hTERT test was positive in 47 out of 60 patients suffering from low-grade tumors (78.3%) and negative in the rest of the 13 patients (21.7%). It was also positive in 18 out of 20 patients suffering from high-grade tumors (90%) and negative in the rest of the 2 patients (10%). Overall, there were not any statistically significant differences between the positive results of hTERT, KRT7, and SVV tests in high-grade and low-grade tumors (p > 0.5).

**Table 4:** The positivity rate of urine cytology, hTERT, KRT7, and SVV in different grades of bladder tumors

Tumor stage	Cytology		hTERT		KRT7		SSV	
	Neg%	Pos%	Neg%	Pos%	Neg%	Pos%	Neg%	Pos%
Low (N: 60)	51 (85.0%)	9 (15.0%)	13 (21.7%)	47 (78.3%)	6 (10.0%)	54 (90.0%)	19 (31.7%)	41 (68.3%)
High (N: 20)	4 (20.0%)	16 (80.0%)	2 (10.0%)	18 (90.0%)	1 (5.0%)	19 (95.0%)	3 (15.0%)	17 (85.0%)
Total (N: 80)	55 (68.8%)	25 (31.3%)	15 (18.7%)	65 (81.3%)	7 (8.7%)	73 (91.3%)	22 (27.5%)	58 (72.5%)
	25/80 (31.3%)		65/80 (81.3%)		73/80 (91.3%)		58/80 (71.3%)	
p value	0.000		0.333		0.673		0.247

### Comparison of hTERT, KRT7, and SVV detection in the different stages of tumors

**Fig. 3:**
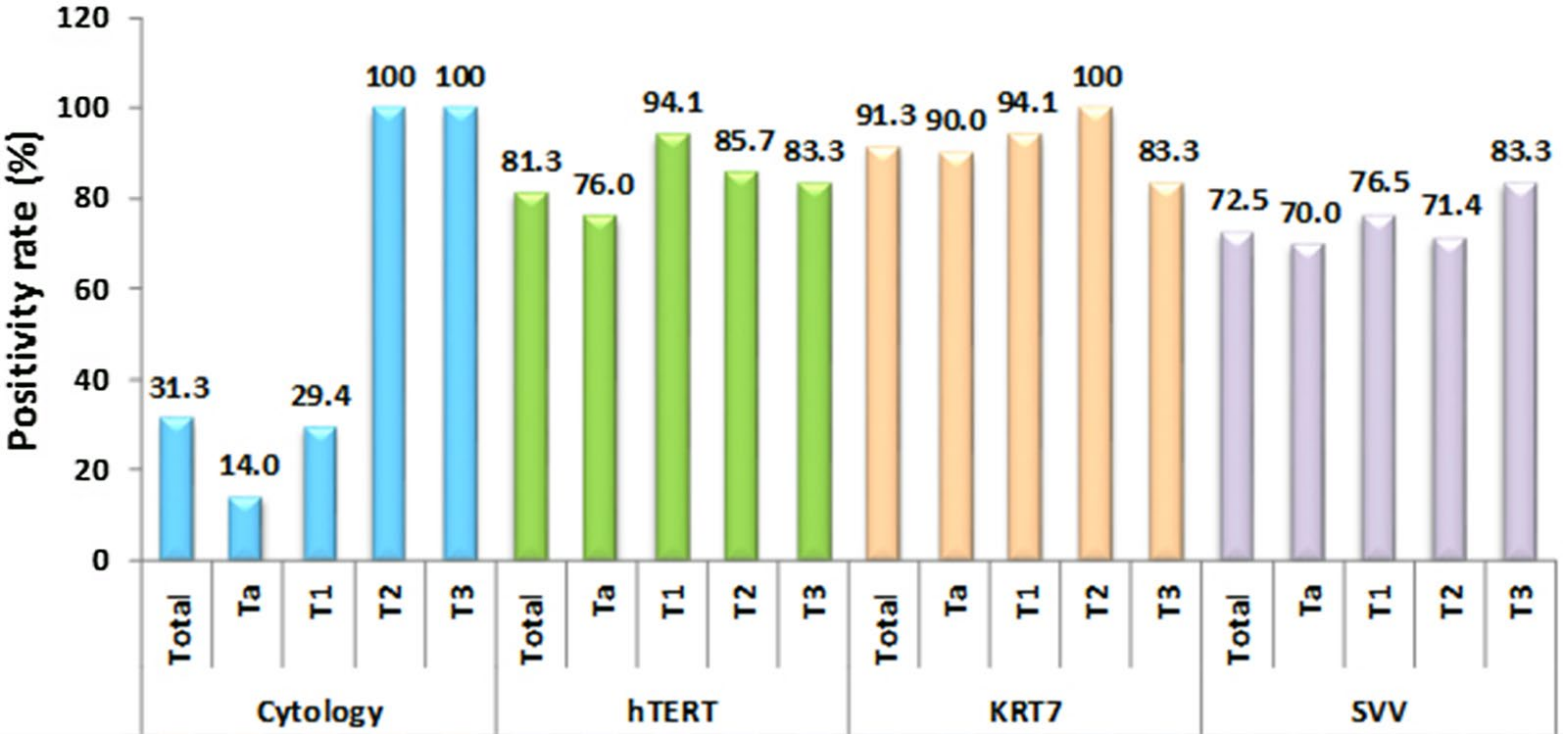
The positivity rate of urine cytology, hTERT, KRT7, and SVV in the different stages of bladder cancer

As shown in Table 5; Fig. 3, when studying the different stages of the tumor, urine cytology was negative in 43 out of 50 patients suffering from stage Ta tumors (86.0%) and was just positive in 7 patients (14%). In 5 out of 17 patients suffering from stage T1, urine cytology was positive in 5 (29.4%), and it was negative in the rest of the 12 patients (70.6%). Urine cytology was positive for both T2 and T3 tumor stages in all 7 and 6 patients, respectively. These results show that with an increase in the stage of the tumor, we have a statistically significant increase in the sensitivity of urine cytology for the diagnosis of bladder carcinoma (p < 0.001). However, there was no statistically significant difference between the results of hTERT, KRT7, and SVV tests and the stages of the tumors (p > 0.5).

**Table 5:** The positivity rate of urine cytology, hTERT, KRT7, and SVV in different stages of bladder tumor

Tumor stage	Cytology		hTERT		KRT7		SSV	
	Neg%	Pos%	Neg%	Pos%	Neg%	Pos%	Neg%	Pos%
Ta (N: 50)	43 (86.0%)	7 (14.0%)	12 (24.0%)	38 (76.0%)	5 (10.0%)	45 (90.0%)	15 (30.0%)	35 (70.0%)
T1 (N: 17)	12 (70.6%)	5 (29.4%)	1 (5.9%)	16 (94.1%)	1 (5.9%)	16 (94.1%)	4 (23.5%)	13 (76.5%)
T2 (N: 7)	0 (0%)	7 (100%)	1 (14.3%)	6 (85.7%)	0 (0%)	7 (100%)	2 (28.6%)	5 (71.4%)
T3 (N: 6)	0 (0%)	6 (100%)	1 (16.7.0%)	5 (83.3%)	1 (16.7%)	5 (83.3%)	1 (16.7%)	5 (83.3%)
Total (N: 80)	56 (70.0%)	25 (31.3%)	15 (18.8%)	65 (81.3%)	7 (8.8%)	73 (91.3%)	22 (27.5%)	58 (72.5%)
	25/80 (31.3%)		65/80 (81.3%)		73/80 (91.3%)		58/80 (72.5%)	
p value	0.000		0.329		0.903		0.507

## Discussion

For a long time, the combination of cystoscopy with urine cytology has constructed the gold standard method for the detection and surveillance of bladder cancer. However, the application of these methods in the clinical platform has been restricted due to some inextricable limitations [8]. Concerning cystoscopy, the invasiveness and cost of the whole process are prime reasons for the decline in its preference for diagnosis. [18]. On the other hand, urine cytology, which is an important noninvasive technique with high specificity, has a restricted application due to its interpreter-dependent low sensitivity for low-grade tumors [19]. While this technique usually performs well with high-stage tumors (T2–T3), the sensitivity of urine cytology for the detection of early-stage tumors is relatively low with a range of 20–40%. This is probably because the Ta–T1 tumors shed fewer cancer cells into the urine [20, 21]. Hence, these challenges highlight the importance of the identification of new urine-based markers for the early as well as appropriate detection of bladder cancer, which is a well-known malignancy with a complicated molecular nature.

There are several FDA-approved immunochromatographic assays for bladder cancer detection. These tests include the measurement of soluble proteins such as bladder tumor-associated antigen (BTA), nuclear matrix protein 22 (NMP22), proteins detected on fixed urothelial cells (ImmunoCyt), and chromosomal aberrations detected by fluorescence in situ hybridization (UroVysion) [22]. Several molecular markers have been recently proposed to improve the diagnostic sensitivity of the urine. Among a wide variety of genes participating in the pathogenesis of human cancers, survivin (SVV) is one of the most studied ones due to its unique characteristics that not only plays a crucial role in the tumor progression but also are profoundly involved in the development of drug resistance. SVV is a member of the inhibitor of apoptosis proteins (IAPs) gene family. Its overexpression inhibits extrinsic and intrinsic pathways of apoptosis. Overexpression of SVV has been reported in almost all human malignancies, including, bladder cancer, lung cancer, breast cancer as well as stomach, esophagus, liver, ovarian and hematological cancers [23, 24]. Moreover, its use as a tumor biomarker has been well-established in some studies on different human cancers [25–27]. However, when it comes to bladder cancer, there are some concerns regarding its efficacy for early as well accurate detection of this type of cancer. The high expression of urinary SVV has been reported in bladder cancer [28]. Despite the positive correlation between the expression of SVV and the malignant degree of bladder cancer [29], conflicting results are reflecting the sensitivity of this gene for the detection of low-grade tumors. In this present study, by measuring the sensitivity and specificity of SVV in the urine specimens of bladder cancer patients using qRT-PCR analysis, we found a performance of 72.5% sensitivity, 90% specificity, and 78.3% accuracy. Notably, this technique was successful in the detection of low-grade bladder cancer patients with a sensitivity of 68.3%; suggesting that the measurement of SVV mRNA in urine may be useful for the detection of bladder cancer in both low- and high-grade cases.

There has been an increasing amount of attention focusing on the role of telomerase in the detection of bladder cancer [30]. Because of its expression in cancer and not in the normal tissues, urine telomerase is a suitable molecular marker for the detection of bladder cancer [31]. The catalytic subunit (hTERT) of telomerase is correlated with the telomerase activity [32]. In similarity with SVV, we found that hTERT, as a single biomarker, could provide an accuracy rate of 87.5% for the diagnosis of bladder cancer, thus, introducing this gene as another valuable prognostic biomarker for this malignancy. The well-established association between SVV and hTERT in the pathogenesis of a wide variety of human cancers proposes a possibility that the co-expression of these genes could serve as a novel biomarker for the early detection of this cancer. Notably, considering both the expression levels of SVV and hTERT in the patients resulted in an accuracy rate up to 92.5%. By combining these two biomarkers, suggests the successfulness of using these combinations in the diagnostics of bladder cancer.

KRT7, a member of the cytokeratin family, is highly expressed in a wide variety of human cancers such as ovarian cancer [33]and squamous cell carcinomas [34]. Recent studies using gene expression profiling of noninvasive primary urothelial tumors such as urothelial neoplasia (stage Ta) biopsies show that KRT7 is an early change in gene expression, which is found to be highly increased when compared to normal biopsies. Consequently, they suggested this expression could be detected in the urine sediments of bladder tumor patients [35]. In another study, the expression level of this gene in the circulating cells of patients undergoing radical cystectomy for urothelial cancer was linked to an increased risk of cancer recurrence and death [36]. Consistently, we found that the sensitivity of KRT7 detection for both low- and high-grade tumors was respectively 90 and 95%, as compared to the 15 and 80% obtained from cytology. Moreover, the sensitivity of KRT7 detection for the early stages of the tumor (Ta and T1) was as high as for the late-stage of tumors (T2 and T3). More interestingly, by combining the results of KRT7 expression and hTERT, a sensitivity of 100% was achieved for bladder cancer patients. Conclusively, our results also showed that combining the expression results of all three genes for bladder cancer patients, either alone or in combination with cytology, provided a sensitivity and NPV of 100%, which was not obtained from any other investigations. Given the significant role of SVV, hTERT, and KRT7 in bladder cancer detection, we suggest that the simultaneous expression analysis of the aforementioned genes could go hand in hand with cytology to provide a better outlook for both the early and accurate diagnosis of patients with bladder cancer in the clinical practice.

## Conclusions

In the present study, we investigated the diagnostic potential of measuring the urinary levels of hTERT, SVV, and KRT7 mRNA for the detection of bladder cancer. The detection of these markers in voided urine samples demonstrates superior sensitivities over cytology. The combined use of these markers offers a powerful potential assay and promising tool for a sensitive, noninvasive, and highly specific method for diagnosis and follow-up of low-grade TCC of the bladder.

## Data Availability

Records and data pertaining to this study are in the patient’s secure medical records in the Department of Urology, Shariati Hospital, Tehran University of Medical Sciences. The datasets generated and analyzed during the current study are not publicly available due to patient privacy issues but the records and raw data could be accessed from RY (r.yahyazadeh@gmail.com) and processed data from the corresponding author on reasonable request (shghaffari2000@yahoo.com).
